# Supplementary material for: From sequence to enzyme mechanism using multi-label machine learning
Source: BMC Bioinformatics. 2014 May 19;15:150. doi: 10.1186/1471-2105-15-150 (PMC4229970; doi:10.1186/1471-2105-15-150)
Supplement: Additional file 2 — Java code of ml2db. Additional file ml2db_code.tar.gz contains the Java source code to run the multi-label machine learning experiments and save the results to database. The code’s Javadoc is included. [file 1471-2105-15-150-S2.zip › additional file 2/ml2db/ml2db/doc/index-files/index-20.html]

T-Index


JavaScript is disabled on your browser.


- Overview
- Package
- Class
- Use
- Tree
- Deprecated
- Index
- Help

- Prev Letter
- Next Letter

- Frames
- No Frames

- All Classes

A B C D E F G H I J K L M N O P Q R S T U V W X 


## T

TABLE\_FOUR\_NAME - Static variable in class test.ml2db.dataharness.DataTableFourTest


TABLE\_NAME - Static variable in class main.ml2db.weka.ArffRecordsTable


TABLE\_ONE\_NAME - Static variable in class test.ml2db.dataharness.DataTableOneTest


TABLE\_THREE\_NAME - Static variable in class test.ml2db.dataharness.DataTableThreeTest


TABLE\_TWO\_NAME - Static variable in class test.ml2db.dataharness.DataTableTwoTest


test() - Method in class test.ml2db.dataharness.DataTableFourTest


test.ml2db - package test.ml2db


test.ml2db.al - package test.ml2db.al


test.ml2db.al.utility.confidence - package test.ml2db.al.utility.confidence


test.ml2db.al.utility.globaldistance - package test.ml2db.al.utility.globaldistance


test.ml2db.al.utility.hybrid - package test.ml2db.al.utility.hybrid


test.ml2db.al.utility.neighbourhood - package test.ml2db.al.utility.neighbourhood


test.ml2db.al.utility.pickers - package test.ml2db.al.utility.pickers


test.ml2db.alltests - package test.ml2db.alltests


test.ml2db.dataharness - package test.ml2db.dataharness


test.ml2db.graphviz - package test.ml2db.graphviz


test.ml2db.mulan.attributesfilter - package test.ml2db.mulan.attributesfilter


test.ml2db.mulan.classifier.lazy - package test.ml2db.mulan.classifier.lazy


test.ml2db.mulan.learn - package test.ml2db.mulan.learn


test.ml2db.mulan.learn.database - package test.ml2db.mulan.learn.database


test.ml2db.mulan.learn.traintest - package test.ml2db.mulan.learn.traintest


test.ml2db.mulan.measuremetrics - package test.ml2db.mulan.measuremetrics


test.ml2db.mulan.mulanarff - package test.ml2db.mulan.mulanarff


test.ml2db.mulan.mulanarff.database - package test.ml2db.mulan.mulanarff.database


test.ml2db.mulan.predict - package test.ml2db.mulan.predict


test.ml2db.weka - package test.ml2db.weka


TEST\_ARFF\_ID - Static variable in class main.ml2db.mulan.learn.crosseval.EvaluationResultsTable
:   the table column to store the arff file id

TEST\_ARFF\_PROPS\_1 - Static variable in class test.ml2db.dataharness.TestProjectParameters


TEST\_ARFF\_PROPS\_2 - Static variable in class test.ml2db.dataharness.TestProjectParameters


TEST\_FIGURES\_PATH - Static variable in class test.ml2db.dataharness.TestProjectParameters


TEST\_MACHINE\_LEARNING\_PROPS - Static variable in class test.ml2db.dataharness.TestProjectParameters


TEST\_REMOTE\_MACHINE\_LEARNING\_PROPS - Static variable in class test.ml2db.dataharness.TestProjectParameters


TEST\_ROLE - Static variable in class test.ml2db.mulan.attributesfilter.AttFiltArffTest


testActiveLearningResultsToDatabase() - Method in class test.ml2db.al.ActiveLearningDatabaseTest


testActiveLearningTestsetStepByStep() - Method in class test.ml2db.al.utility.neighbourhood.NeighbourhoodExperimentDetailsTest


testAddAndFindADistance() - Method in class test.ml2db.al.utility.globaldistance.DistancesStorageTest


testAddAndGetLabDistances() - Method in class test.ml2db.al.utility.globaldistance.DistancesStoreTest


testANewFilteredFileContent(int, int, String[]) - Static method in class test.ml2db.mulan.attributesfilter.AttFiltArffTest


testArffName() - Method in class test.ml2db.mulan.attributesfilter.AttFiltArffTest


testArffWithNumericAttributes() - Method in class test.ml2db.weka.ArffTest


testCalculateAllDistances() - Method in class test.ml2db.al.utility.globaldistance.DistancesCalculatorTest
:   Test method for
    `DistancesCalculator.calculateAllDistances()`
    .

testCheckTrainTestEvalRow(TableRow) - Static method in class test.ml2db.mulan.learn.traintest.TrainTestEvaluatorTest
:   Executes generic tests (number of metrics etc.) on a table row containing
    evaluation results.

testCreateAttribute() - Method in class test.ml2db.weka.WekaTest


testCreateAttributeFactory() - Method in class test.ml2db.mulan.attributesfilter.AttFiltDataSetGeneratorTest


testCreateAttributesList() - Method in class test.ml2db.mulan.mulanarff.MulanDataSetGeneratorTest


testCreateAttributeVector() - Method in class test.ml2db.weka.WekaTest


testCreateInstance() - Method in class test.ml2db.weka.WekaTest


testCreateInstances() - Method in class test.ml2db.weka.WekaTest


testCreateNewTestArff() - Method in class test.ml2db.mulan.attributesfilter.AttFilterTest


testCrossValidate1() - Method in class test.ml2db.mulan.learn.CrossEvaluatorTest


testCrossValidate2() - Method in class test.ml2db.mulan.learn.CrossEvaluatorTest


testDatabase() - Method in class test.ml2db.dataharness.DatabaseTest


testDataStatistics() - Method in class test.ml2db.mulan.mulanarff.MulanDataSetGeneratorTest


testDataStatistics() - Method in class test.ml2db.weka.DataSetWriterTest


testDeserialize() - Method in class test.ml2db.mulan.mulanarff.SerializerTest


testDistanceInstanceInstance() - Method in class main.ml2db.weka.JaccardDistanceTest
:   Test method for
    `JaccardDistanceStored.distance(weka.core.Instance, weka.core.Instance)`
    .

testEvaluation1() - Method in class test.ml2db.mulan.learn.traintest.TrainTestEvaluatorTest


testEvaluationResults1(TableRow) - Static method in class test.ml2db.mulan.learn.traintest.TrainTestEvaluatorTest


testFillInstances() - Method in class test.ml2db.mulan.mulanarff.MulanInstancesFillerTest


testFillNonExistingInstances() - Method in class test.ml2db.mulan.mulanarff.MulanInstancesFillerTest


testFindByHashCode() - Method in class test.ml2db.al.utility.neighbourhood.NeighbourhoodNetworkTest


testGenerateArffFileFromDb2() - Method in class test.ml2db.mulan.mulanarff.MulanArffTest
:   public static String getExpectedMulanArff1() { return
    "@relation test\_data\n\n" + MulanArffTest.ATT1\_TO\_STRING + "\n" +
    MulanArffTest.ATT2\_TO\_STRING + "\n" + MulanArffTest.ATT3\_TO\_STRING + "\n"
    + MulanArffTest.ATT4\_TO\_STRING + "\n" + MulanArffTest.ATT5\_TO\_STRING +
    "\n" + "@attribute class1 {0,1}\n" + "@attribute class2 {0,1}\n" +
    "@attribute class3 {0,1}\n" + "@attribute class4 {0,1}\n" + "\n@data\n" +
    "% " + DataOne.INST1 + "\n" + MulanArffTest.arffInstance1 + "\n" + "% " +
    DataOne.INST2 + "\n" + MulanArffTest.arffInstance2 + "\n" + "% " +
    DataOne.INST4 + "\n" + MulanArffTest.arffInstance4 + "\n" + "% " +
    DataOne.INSTATTVOID + "\n" + MulanArffTest.arffInstanceAttributeVoid +
    "\n" + "% " + DataOne.INSTCLASSATTVOID + "\n" +
    MulanArffTest.arffInstanceClassAttributeVoid + "\n" + "% " +
    DataOne.INSTCLASSVOID + "\n" + MulanArffTest.arffInstanceClassVoid; }
    public static String getExpectedMulanArff2() { String attributes =
    "@relation test\_ec\_data\n\n@attribute 1.-.-.- {0,1}\n@attribute 1.1.-.- {0,1}\n@attribute 1.1.1.- {0,1}\n@attribute 1.1.1.1 {0,1}\n@attribute 2.-.-.- {0,1}\n@attribute 2.2.-.- {0,1}\n@attribute 2.2.2.- {0,1}\n@attribute 2.2.2.2 {0,1}\n@attribute 3.-.-.- {0,1}\n@attribute 3.3.-.- {0,1}\n@attribute 3.3.3.- {0,1}\n@attribute 3.3.3.3 {0,1}\n@attribute att1 {0,1}\n@attribute att2 {0,1}\n@attribute att3 {0,1}\n@attribute att4 {0,1}\n@attribute att5 {0,1}\n\n"
    ; String instances =
    "@data\n% inst1\n{0 1,1 1,2 1,3 1,12 1}\n% inst10\n{4 1,5 1,6 1,7 1,16 1}\n% inst11\n{4 1,5 1,6 1,7 1}\n% inst12\n{12 1}\n% inst13\n{}\n% inst2\n{4 1,5 1,6 1,7 1,14 1}\n% inst3\n{0 1,1 1,2 1,3 1,12 1}\n% inst4\n{4 1,5 1,6 1,7 1,14 1}\n% inst5\n{0 1,1 1,2 1,3 1,12 1}\n% inst6\n{4 1,5 1,6 1,7 1,14 1}\n% inst7\n{0 1,1 1,2 1,3 1,12 1}\n% inst8\n{4 1,5 1,6 1,7 1,14 1}\n% inst9\n{0 1,1 1,2 1,3 1,15 1}\n% instA\n{8 1,9 1,10 1,11 1,13 1}\n% instB\n{4 1,5 1,6 1,7 1,14 1}\n% instC\n{4 1,5 1,6 1,7 1,13 1,14 1}\n% inst\_att\_void\n{4 1,5 1,6 1,7 1}\n% inst\_class\_att\_void\n{}\n% inst\_class\_void\n{13 1}"
    ;
    return attributes + instances; }

testGenerateBinaryAttribute() - Method in class test.ml2db.weka.AttributeFactoryTest


testGenerateMulanArffNumericAttributes() - Method in class test.ml2db.mulan.mulanarff.MulanArffTest


testGenerateNominalAttribute() - Method in class test.ml2db.weka.AttributeFactoryTest


testGenerateNumericAttribute() - Method in class test.ml2db.weka.AttributeFactoryTest


testGetArff() - Method in class test.ml2db.weka.DataSetManagerTest


testGetAttributes() - Method in class test.ml2db.weka.DataSetGeneratorTest


testGetAverageConfidenceUncertainty() - Method in class test.ml2db.al.utility.confidence.ConfidenceCalculatorTest


testGetAverageUtility() - Method in class test.ml2db.al.utility.confidence.ConfidenceCalculatorTest


testGetBipartitions() - Method in class test.ml2db.mulan.measuremetrics.AnnotationsReaderTest
:   Test method for
    `AnnotationsReader.getPredictedBipartitions()`
    .

testGetColumnDefinitions() - Method in class test.ml2db.weka.ArffPropsTableTest


testGetColumnsList() - Method in class test.ml2db.mulan.learn.EvaluationResultsTableTest


testGetConfidences() - Method in class test.ml2db.al.utility.confidence.ConfidenceCalculatorTest


testGetDataSetName() - Static method in class test.ml2db.weka.DataSetManagerTest


testGetDot() - Method in class test.ml2db.graphviz.GraphVizArffColouredTest


testGetDot1() - Method in class test.ml2db.graphviz.GraphVizArffTest


testGetDot2() - Method in class test.ml2db.graphviz.GraphVizArffTest


testGetDotSource() - Method in class test.ml2db.graphviz.GraphVizTest
:   Test method for `GraphViz.getDotSource()`.

testGetEvaluation() - Method in class test.ml2db.mulan.measuremetrics.PredictionsPerformanceMeasurerTest


testGetExampleBasedAccuracyMeasure() - Method in class test.ml2db.mulan.learn.EvaluationParametersTest


testGetFeaturesByDescendentFrequency() - Method in class test.ml2db.al.utility.pickers.AttSetByFrequencyPickerTest
:   Test method for
    `ByFrequencyPicker.getFeaturesByDescendentFrequency()`
    .

testGetFilteredTestArffRecord() - Method in class test.ml2db.mulan.learn.traintest.TrainTestExperimenterTest


testGetFreqToInstancesMap() - Method in class test.ml2db.al.utility.neighbourhood.NearestNeighboursByFreqUtilityTest


testGetFreqToInstancesMap2() - Method in class test.ml2db.al.utility.neighbourhood.NearestNeighboursByFreqUtilityTest


testGetGraph() - Method in class test.ml2db.graphviz.GraphVizTest
:   Test method for
    `GraphViz.getGraphImage(java.lang.String, java.lang.String)`
    .

testGetInstances() - Method in class test.ml2db.al.utility.neighbourhood.NeighbourhoodNetworkTest


testGetInstances() - Method in class test.ml2db.mulan.measuremetrics.AnnotationsReaderTest
:   Test method for
    `AnnotationsReader.getInstances()`
    .

testGetInstancesAndLabels() - Method in class test.ml2db.mulan.measuremetrics.AnnotationsReaderTest
:   Test method for
    `AnnotationsReader.getPredictionsInstancesAndLabels()`
    .

testGetInstancesNamesFromFile() - Method in class test.ml2db.mulan.mulanarff.database.MulanArffRecordTest


testGetInstanceToFeatureMap() - Method in class test.ml2db.al.utility.neighbourhood.NearestNeighboursByFreqUtilityTest


testGetInstanceToFeatureMap2() - Method in class test.ml2db.al.utility.neighbourhood.NearestNeighboursByFreqUtilityTest


testGetLabelledDistances() - Method in class test.ml2db.al.utility.globaldistance.GlobalDistanceMetricTest


testGetLabelledDistancesTrainset() - Method in class test.ml2db.al.utility.globaldistance.GlobalDistanceMetricTest


testGetLabels() - Method in class test.ml2db.mulan.measuremetrics.AnnotationsReaderTest
:   Test method for
    `AnnotationsReader.getLabels()` .

testGetLearnerBaseType() - Method in class test.ml2db.mulan.learn.MulanLearnerTest


testGetLearnerType() - Method in class test.ml2db.mulan.learn.MulanLearnerTest


testGetLernerFullName() - Method in class test.ml2db.mulan.learn.MulanLearnerTest


testGetLinearNNSearch() - Method in class test.ml2db.al.utility.neighbourhood.NearestNeighboursByFreqUtilityTest
:   Test method for
    `NearestNeighboursByFreqUtility.getLinearNNSearch()`
    .

testGetMeasures() - Method in class test.ml2db.mulan.learn.EvaluationParametersTest


testGetMetricName() - Method in class test.ml2db.al.utility.confidence.ConfidenceMetricTest


testGetMetricName() - Method in class test.ml2db.al.utility.hybrid.GlobalDistanceWithConfidenceMetricTest
:   Test method for
    `GlobalDistanceWithConfidenceMetric.getMetricName()`
    .

testGetMLOutput() - Method in class test.ml2db.mulan.measuremetrics.AnnotationsReaderTest


testGetNeighbours() - Method in class test.ml2db.al.utility.neighbourhood.LinearNNSearchSkipInstanceItselfTest
:   Test method for
    `main.ml2db.al.utility.neighbours.LinearNNSearchSkipInstanceItself#getNeighbours(weka.core.Instance, uk.ac.ed.inf.enzml.mulan.mulanarff.IdMultiLabelInstances, int, boolean)`
    .

testGetNumInstances() - Method in class test.ml2db.mulan.mulanarff.StatisticsTest
:   Test method for
    `Statistics.getNumInstances()`.

testGetOptions() - Method in class test.ml2db.weka.AttributeFactoryTest


testGetPositioning() - Method in class test.ml2db.al.utility.globaldistance.GlobalDistanceMetricTest


testGetProperties1() - Method in class test.ml2db.dataharness.ArffPropsOneTest


testGetProperties1() - Method in class test.ml2db.dataharness.ArffPropsTwoTest


testGetPropertiesFilePath1() - Method in class test.ml2db.dataharness.ArffPropsOneTest


testGetPropertiesFilePath2() - Method in class test.ml2db.dataharness.ArffPropsTwoTest


testGetProperty1ValueFromTable() - Method in class test.ml2db.weka.ArffPropsTableTest


testGetStatistics() - Method in class test.ml2db.weka.DataSetGeneratorTest


testGetTableField() - Method in class test.ml2db.mulan.learn.EvaluationResultsTableTest


testGetTestInstanceAndNeighboursMap() - Method in class main.ml2db.mulan.mulanarff.NeighboursPrinterTest
:   Test method for
    `NeighboursPrinter.getInstanceAndNeighboursMap()`
    .

testGetTrainInstanceAndNeighboursMap() - Method in class main.ml2db.mulan.mulanarff.NeighboursPrinterTest
:   Test method for
    `NeighboursPrinter.getInstanceAndNeighboursMap()`
    .

testGetUncertainty() - Method in class test.ml2db.al.utility.confidence.ConfidenceCalculatorTest


testGetUnlabelledDistances() - Method in class test.ml2db.al.utility.globaldistance.GlobalDistanceMetricTest


testInitialisationKO() - Method in class test.ml2db.weka.DataSetCheckerTest
:   No xml query property, the initialisation should fail \*before\* generating
    the arff and xml files (and not waste time in generating the arff file
    and then fail at the generation of the xml file)

testInitialisationOK() - Method in class test.ml2db.weka.DataSetCheckerTest


testInstanceAttributeValueIsInTable(String, String, String) - Method in class test.ml2db.dataharness.DataTableTest


testInstancesSection() - Method in class test.ml2db.weka.DataSetWriterTest


testJoinInstances() - Method in class test.ml2db.al.utility.globaldistance.DistancesStorageTest


testKNearestNeighboursInstanceIntBoolean() - Method in class test.ml2db.al.utility.neighbourhood.LinearNNSearchSkipInstanceItselfTest
:   Test method for
    `LinearNNSearchSkipInstanceItself.kNearestNeighbours(weka.core.Instance, int, boolean)`
    .

testLinearNNSearchSkipInstanceItself() - Method in class test.ml2db.al.utility.neighbourhood.LinearNNSearchSkipInstanceItselfTest
:   Test method for
    `main.ml2db.al.utility.neighbours.LinearNNSearchSkipInstanceItself#LinearNNSearchSkipInstanceItself(uk.ac.ed.inf.enzml.mulan.mulanarff.IdMultiLabelInstances)`
    .

testLoadDataMulan() - Method in class test.ml2db.mulan.mulanarff.MulanDataSetDbLoaderTest


testLoadDataTableOne() - Method in class test.ml2db.weka.DataSetDbLoaderTest


testLogHeader() - Method in class test.ml2db.weka.DataSetWriterTest


testLogTail() - Method in class test.ml2db.weka.DataSetWriterTest


testMetrics\_Train\_1\_2\_empty\_Test\_3(TableRow) - Static method in class test.ml2db.mulan.learn.traintest.EvaluationMetricsTest


testMetrics\_Train\_1\_2\_Test\_3(TableRow) - Static method in class test.ml2db.mulan.learn.traintest.EvaluationMetricsTest


testMetricsTrain\_1\_2\_Test\_1\_2(TableRow) - Static method in class test.ml2db.mulan.learn.traintest.EvaluationMetricsTest


testNeedsNominalOrNumericAttributes() - Method in class test.ml2db.weka.InstAttValueMapTest


testNewTestsetFile() - Method in class test.ml2db.mulan.attributesfilter.AttFiltArffTest


testNewTrainsetFile() - Method in class test.ml2db.mulan.attributesfilter.AttFiltArffTest


testPredictions() - Method in class test.ml2db.mulan.predict.PredictionsSaverTest


testPredictorI1I2\_vs\_I1I2() - Method in class test.ml2db.mulan.predict.PredictionsSaverTest


testPredictorI3Empty\_vs\_I1I2() - Method in class test.ml2db.mulan.predict.PredictionsSaverTest


testPredictorTrainTest() - Method in class test.ml2db.mulan.predict.PredictionsSaverTest


testPrepareQueryInstancesList() - Method in class test.ml2db.al.utility.pickers.LabelsByFreqPickerTest


testPrepareQueryInstancesList() - Method in class test.ml2db.al.utility.pickers.RandomAttributePickerTest


testPrepareQueryInstancesList() - Method in class test.ml2db.al.utility.pickers.RandomAttributeSetPickerTest


testPrintColourGraphviz() - Method in class test.ml2db.al.GraphVizPrinterTest


testPrintGraphviz() - Method in class test.ml2db.al.GraphVizPrinterTest


TestProjectParameters - Class in test.ml2db.dataharness


TestProjectParameters() - Constructor for class test.ml2db.dataharness.TestProjectParameters


testPropsFileIsReadable(String) - Method in class test.ml2db.dataharness.ArffPropsFilesTest


testPropsFiles() - Method in class test.ml2db.dataharness.ArffPropsFilesTest


testQueries() - Method in class test.ml2db.dataharness.ArffPropsQueriesOneTest


testQueries(ArffPropsFromFile, String, String, String) - Static method in class test.ml2db.dataharness.ArffPropsQueriesTest
:   Test queries

testQueries() - Method in class test.ml2db.dataharness.ArffPropsQueriesTwoTest


testQueryResults(String, int, Vector<String>) - Static method in class test.ml2db.dataharness.ArffPropsQueriesTest


testReadSource() - Method in class test.ml2db.graphviz.GraphVizTest
:   Test method for
    `GraphViz.readSource(java.lang.String)` .

testRun() - Method in class test.ml2db.mulan.learn.ExperimenterTest


testRunningTime() - Method in class test.ml2db.mulan.learn.CrossEvaluatorTest
:   public void testEvaluationResults() { MulanEvaluator evaluator =
    MulanEvaluatorTest.getMulanEvaluator(); assertFalse(evaluator == null);
    if (evaluator != null) { Evaluation[] results =
    evaluator.getEvaluationResults(); assertEquals(10, results.length);
    Evaluation e0 = results[0]; System.out.println(e0.toString());
    java.util.List measures = e0.getMeasures(); assertEquals(22,
    measures.size());
    // hamming loss Measure m0 = measures.get(0);
    assertEquals("Hamming Loss", m0.getName()); double value = m0.getValue();
    System.out.println("MulanEvaluatorTest.

testSaveProperties1ToTable() - Method in class test.ml2db.weka.ArffPropsTableTest


testSaveResults() - Method in class test.ml2db.mulan.learn.EvaluationResultsTableTest


testSerializeMlkNN2() - Static method in class test.ml2db.mulan.mulanarff.SerializerTest


testSerializeObject() - Method in class test.ml2db.al.utility.globaldistance.DistancesCalculatorTest
:   Test method for
    `main.ml2db.Serializer#serialize(java.lang.Object, java.lang.String)`
    .

TESTSET\_LABELS - Static variable in class main.ml2db.mulan.learn.crosseval.EvaluationResultsTable


testSetAttributeValue() - Method in class test.ml2db.weka.WekaTest


testSetClass() - Method in class test.ml2db.weka.WekaTest


testStats1() - Method in class test.ml2db.al.StatisticsTest


testTableContent() - Method in class test.ml2db.dataharness.DataTableOneTest


testTableContent() - Method in class test.ml2db.dataharness.DataTableThreeTest


testTableContent() - Method in class test.ml2db.dataharness.DataTableTwoTest


testTableInstanceAttribute(String, String) - Method in class test.ml2db.dataharness.DataTableTest


testTableInstanceClass(String, String) - Method in class test.ml2db.dataharness.DataTableTest


testTestSet() - Method in class test.ml2db.mulan.mulanarff.IdMultiLabelInstancesTest


testToFullString() - Method in class test.ml2db.mulan.mulanarff.IdInstanceTest


testWriteArff() - Method in class test.ml2db.mulan.mulanarff.MulanDataSetManagerTest


testWriteInstance() - Method in class test.ml2db.weka.InstancesFillerTest
:   public void testFillNonExistingInstances() { InstancesFactory factory =
    InstancesFactoryTest.instancesFactory(); SparseInstance instance = new
    SparseInstance(factory.getDataSet() .getAttributes().size());
    // set the dataset (Instances) for the instance
    instance.setDataset(factory.getDataSet().getInstances());
    // fill instance attribute values instance =
    factory.fillInstance("empty", instance);
    assertEquals("@attribute att1 {n,y}", instance.attribute(0).toString());
    assertEquals("@attribute att3 {n,y}", instance.attribute(2).toString());
    assertEquals(4, instance.classIndex()); assertEquals("NaN",
    Double.toString(instance.classValue())); assertEquals(4,
    instance.index(0)); assertEquals(5, instance.numAttributes());
    assertEquals(1, instance.numValues()); assertEquals("{4 ?}",
    instance.toString());
    }

testWriteOnDatabase() - Method in class test.ml2db.weka.ArffPropsTableTest


TIMESTAMP\_FIELD - Static variable in class main.ml2db.weka.ArffPropsFromFile
:   the timestamp field

toGif(String, String) - Method in class main.ml2db.graphviz.GraphViz
:   Writes this object dot graph to GIF image in the desired directory

toGifToTmp(String) - Method in class main.ml2db.graphviz.GraphViz
:   Writes this object dot graph to GIF image in the \tmp\ directory

toIdString() - Method in class main.ml2db.mulan.mulanarff.IdInstance
:   Includes the attributes names.

toImage(String, String, String) - Method in class main.ml2db.graphviz.GraphViz
:   Writes this object dot graph to the desired image format

toImageFile(String, String) - Method in class main.ml2db.graphviz.GraphViz
:   Writes this object dot code to image file

toJpg(String, String) - Method in class main.ml2db.graphviz.GraphViz
:   Writes this object dot graph to GIF image in the desired directory

toPdf(String, String) - Method in class main.ml2db.graphviz.GraphViz


topPhiCorrelatedLabels(int, int) - Method in class main.ml2db.mulan.mulanarff.Statistics
:   Returns the indices of the labels that have the strongest phi correlation
    with the label which is given as a parameter.

toSparseAttributesString() - Method in class main.ml2db.mulan.mulanarff.IdInstance


toString() - Method in class main.ml2db.mulan.learn.MulanLearner


toString() - Method in class main.ml2db.mulan.learn.traintest.TrainTestExperimenterSerialized


toString() - Method in class main.ml2db.mulan.mulanarff.IdInstance


toString() - Method in class main.ml2db.mulan.mulanarff.IdMultiLabelInstances


toString() - Method in class main.ml2db.mulan.mulanarff.Statistics
:   returns various multilabel statistics in textual representation

toUndirectedGraphvizString() - Method in class main.ml2db.mulan.mulanarff.NeighboursPrinter


TRAIN\_ARFF\_ID - Static variable in class main.ml2db.mulan.learn.crosseval.EvaluationResultsTable
:   the table column to store the arff file id

TRAIN\_ROLE - Static variable in class test.ml2db.mulan.attributesfilter.AttFiltArffTest


trainAndSerialize(int, MulanLearner, DbManagers, String) - Static method in class main.ml2db.mulan.learn.traintest.TrainTestExperimenterSerialized


TrainTestEvaluator - Class in main.ml2db.mulan.learn.traintest
:   Train/test evaluator.

TrainTestEvaluator(TrainTestExperimenter) - Constructor for class main.ml2db.mulan.learn.traintest.TrainTestEvaluator


TrainTestEvaluatorSerialised - Class in main.ml2db.mulan.learn.traintest
:   Class

TrainTestEvaluatorSerialised(TrainTestExperimenter, String) - Constructor for class main.ml2db.mulan.learn.traintest.TrainTestEvaluatorSerialised


TrainTestEvaluatorTest - Class in test.ml2db.mulan.learn.traintest


TrainTestEvaluatorTest() - Constructor for class test.ml2db.mulan.learn.traintest.TrainTestEvaluatorTest


TrainTestExperimenter - Class in main.ml2db.mulan.learn.traintest
:   Using Mulan (multi-label machine learning), this class initialises and
    executes Train-Test evaluation (two arff records are needed, for the training
    and testing files) (calling `TrainTestEvaluator`).

TrainTestExperimenter(DbManagers, int, int, MulanLearner, String) - Constructor for class main.ml2db.mulan.learn.traintest.TrainTestExperimenter


TrainTestExperimenter(IdMultiLabelInstances, IdMultiLabelInstances, DbManagers, MulanLearner, String) - Constructor for class main.ml2db.mulan.learn.traintest.TrainTestExperimenter


TrainTestExperimenterSerialized - Class in main.ml2db.mulan.learn.traintest
:   Executes a train/test evaluation using a previously trained model saved on
    file.

TrainTestExperimenterSerialized(DbManagers, int, int, MulanLearner, String, String) - Constructor for class main.ml2db.mulan.learn.traintest.TrainTestExperimenterSerialized


TrainTestExperimenterTest - Class in test.ml2db.mulan.learn.traintest


TrainTestExperimenterTest() - Constructor for class test.ml2db.mulan.learn.traintest.TrainTestExperimenterTest


TrainTestFullRun - Class in main.ml2db.mulan.learn.traintest
:   Runs a full train/test evaluation: 1.

TrainTestFullRun() - Constructor for class main.ml2db.mulan.learn.traintest.TrainTestFullRun


TrainTestInputManager - Class in main.ml2db.mulan.learn.traintest
:   An input manager that gets both the training and testing instances from a
    database arff record identifier.

TrainTestInputManager(IdMultiLabelInstances, IdMultiLabelInstances, TrainTestExperimenter) - Constructor for class main.ml2db.mulan.learn.traintest.TrainTestInputManager
:   Gets the instances as object.

TrainTestInputManager(IdMultiLabelInstances, TrainTestExperimenter) - Constructor for class main.ml2db.mulan.learn.traintest.TrainTestInputManager
:   Gets the instances as object.

TrainTestInputManager(int, DbManagers, TrainTestExperimenter) - Constructor for class main.ml2db.mulan.learn.traintest.TrainTestInputManager


TrainTestInputManager(int, int, DbManagers, TrainTestExperimenter) - Constructor for class main.ml2db.mulan.learn.traintest.TrainTestInputManager


TrainTestOutputManager - Class in main.ml2db.mulan.learn.traintest
:   Class

TrainTestOutputManager(DbManager, String, Table, TrainTestExperimenter) - Constructor for class main.ml2db.mulan.learn.traintest.TrainTestOutputManager


trainTestValidate(IdMultiLabelInstances, IdMultiLabelInstances, boolean, boolean) - Method in class main.ml2db.mulan.learn.traintest.TrainTestEvaluator


TRUE\_POSITIVES - Static variable in class main.ml2db.mulan.predict.PredictionsSaver


TruePositives - Class in main.ml2db.mulan.learn.evaluation
:   Class

TruePositives(int) - Constructor for class main.ml2db.mulan.learn.evaluation.TruePositives


truth1 - Static variable in class test.ml2db.mulan.measuremetrics.AnnotationsReaderTest

A B C D E F G H I J K L M N O P Q R S T U V W X

- Overview
- Package
- Class
- Use
- Tree
- Deprecated
- Index
- Help

- Prev Letter
- Next Letter

- Frames
- No Frames

- All Classes
